# TARGETING BIOLOGICAL AGING MECHANISMS OF LOWER URINARY TRACT SYMPTOMS IN OLDER MEN WITH AN EXERCISE INTERVENTION

**DOI:** 10.1093/geroni/igae098.2048

**Published:** 2024-12-31

**Authors:** Scott Bauer

**Affiliations:** University of California San Francisco, San Francisco, California, United States

## Abstract

Physical frailty is associated with lower urinary tract symptoms (LUTS) severity and progression among older men with suspected benign prostatic hyperplasia (BPH). Prospective cohort studies have consistently demonstrated that both physically inactive and frail men are more likely to develop BPH or experience LUTS progression. The combination of aerobic plus resistance exercise has been demonstrated in clinical trials to have pleotropic effects that delay, prevent, or treat many frailty-related conditions that often co-occur with LUTS and BPH, including cardiovascular disease, diabetes, and erectile dysfunction. Although it has never been tested in humans, pre-clinical mouse models of BPH demonstrate improved voiding behaviors after exposure to exercise and exercise is known to improve several mechanisms that are implicated in human LUTS/BPH pathophysiology, including insulin resistance, endocrine dysregulation, and mitochondrial dysfunction. The recently launched PROUD study (NCT06225479; MPIs: Bauer, Kenfield) is a 2-arm pilot randomized controlled trial among physically inactive older men with LUTS/BPH testing the effect of a 12-week remote exercise intervention versus health education control. Outcomes include: 1) feasibility, acceptability, fidelity, and safety; 2) changes in LUTS, urologic and physical function, and physical activity; and 3) feasibility of detecting exercise-induced change in novel frailty-related mechanistic measures. This symposium session will review the scientific rationale, key study design and analytic decisions, enrollment progress thus far, and collaborative opportunities using stored biobank specimens.

